# Microscopic subinguinal varicocelectomy for testicular pain: a retrospective study on outcomes and predictors of pain relief

**DOI:** 10.1186/s12610-020-00119-z

**Published:** 2021-01-07

**Authors:** Abdullah Al-Gadheeb, Hossam S. El-Tholoth, Abdulaziz Albalawi, Abdulmajeed Althobity, Mussa AlNumi, Tala Alafraa, Amr Jad

**Affiliations:** Department of Urology, Prince Sultan Military Medical City, Riyadh, Saudi Arabia

**Keywords:** Pain relief, Testicular pain, Varicocele, Varicocelectomy, Soulagement de la douleur, Douleur testiculaire, Varicocèle, Varicocèlectomie

## Abstract

**Background:**

Approximately 2–10% of patients with varicocele complain of pain. Varicocelectomy for testicular pain is a surgical choice when conservative therapy fails to relieve the pain. Different variables have been reported as prognostic factors for pain relief following varicocele ligation. Moreover, the success rate of varicocelectomy for testicular pain has varied among studies. This retrospective study aimed to investigate the predictors and success rate of microscopic subinguinal varicocelectomy performed for the treatment of painful varicocele.

**Results:**

Among the 132 patients, 83.3% reported pain relief. A significant association was identified between varicocelectomy for unilateral testicular pain and pain resolution (*P* < 0.0001); no other factors were predictors of pain relief.

**Conclusions:**

Microscopic subinguinal varicocelectomy for testicular pain is an effective surgical alternative. Varicocelectomy for unilateral testicular pain may predict postoperative pain relief in appropriately selected patients.

## Résumé

**Contexte:**

Environ 2 à 10 % des patients porteurs de varicocèle se plaignent de douleur. La varicocèlectomie pour douleur testiculaire est un choix chirurgical lorsque le traitement conservateur ne parvient pas à soulager la douleur. Différentes variables ont été rapportées comme facteurs pronostiques du soulagement de la douleur après ligature de la varicocèle. Par ailleurs, le taux de réussite de la varicocèlectomie pour douleur testiculaire varie selon les études. Cette étude rétrospective visait à étudier les facteurs prédictifs et le taux de réussite de la varicocèlectomie subinguinale microscopique réalisée pour traiter une varicocèle douloureuse.

**Résultats:**

Parmi les 132 patients opérés, 83,3% ont rapporté le soulagement de la douleur. Une association significative a été identifiée entre la varicocèlectomie pour douleur testiculaire unilatérale et la résolution de la douleur (*P* <0,0001) ; aucun autre facteur n’était prédictif du soulagement de la douleur.

**Conclusions:**

La varicocèlectomie subinguinale microscopique pour douleur testiculaire est une alternative chirurgicale efficace. La varicocèlectomie pour douleur testiculaire unilatérale peut prédire le soulagement postopératoire de la douleur chez les patients convenablement choisis.

## Background

Varicocele is an abnormal dilatation of the gonadal veins within the pampiniform plexus of the spermatic cord [1]. The prevalence of varicocele varies and is estimated as 15% [2]. Although the etiology of varicocele is multifactorial, two predisposing factors are considered, namely early adolescence and increased intra-abdominal pressure during childhood [3]. Most patients remain asymptomatic; however, the most common clinical presentations are infertility and chronic scrotal pain [1]. Varicocelectomy is usually indicated in patients with infertility, adolescents with testicular hypotrophy, and patients with persistent pain [4]. Approximately 2–10% of patients with varicocele complain of pain, mainly in the inguinal area or scrotum [5], which ranges from dull discomfort to sharp pain and may increase after standing, sitting, or physical exertion [6]. Conservative treatment of varicocele-associated pain, including nonsteroidal anti-inflammatory drugs, scrotal elevation, and limitation of physical activities, can be offered; however, these measures are only successful in a few patients [7]. The rate of pain resolution in conservatively treated men was 4.2–15.2% [8, 9]. For patients who experience persistent pain despite conservative treatment, varicocelectomy is an option [7]. However, its success rate has varied among studies, and different variables have been reported as prognostic factors for pain relief after varicocele ligation [10]. Therefore, we investigated the predictors of pain resolution after varicocelectomy and evaluated the success rate of the procedure.

## Materials and methods

**Fig. 1:**
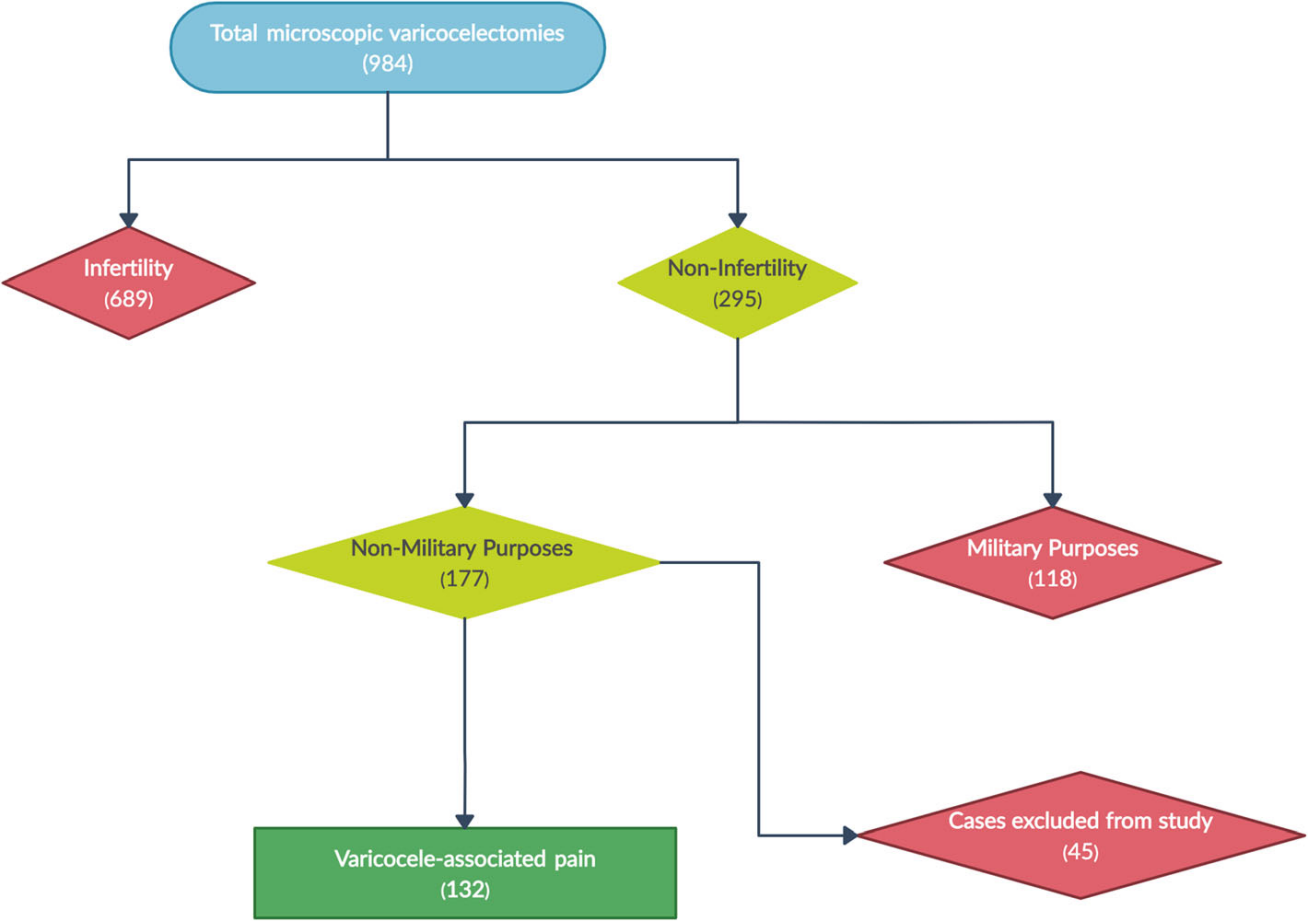
Patient flowchart

This retrospective study was performed by reviewing medical records of the patients. From March 2016 to February 2019, 984 patients underwent microscopic subinguinal varicocelectomy at the Prince Sultan Military Medical City, Riyadh, Saudi Arabia. Of these men, 689 were operated for infertility and 118 for military purposes (varicocele was diagnosed during the prerequisite examination of the military services and varicocelectomy was performed as per military request to re-qualify for military service). Additionally, 45 patients who had varicocele associated with other findings that cause testicular pain, such as sexually transmitted disease, urinary tract infection, prostatitis, testicular torsion, trauma, or history of scrotal surgery, were excluded from the study. The study involved 132 patients who did not have infertility concerns and underwent microscopic subinguinal varicocelectomy for only varicocele-associated testicular pain following failed conservative treatment (limitations in activity, scrotal elevation, and nonsteroidal anti-inflammatory medications) for 3 months (Fig. 1). This study was approved by the Research Ethics Committee (No. 1327), and written informed consent was obtained from all patients. Furthermore, the study was performed according to the Declaration of Helsinki.

A varicocele was diagnosed based on physical examination followed by Doppler ultrasound findings. The grades of varicocele were defined as grade I, only palpable with Valsalva maneuver; grade II, easily palpable without Valsalva maneuver but not visible; and grade III, palpable and visible [11]. All medical records were retrospectively reviewed to document the demographic data of patients (age, body mass index, and smoking status), clinical grading (I, II, or III), maximum dilated vein diameter, the site (unilateral or bilateral) and quality (dragging or dull) of pain before surgery, intraoperative findings, and complications. The severity of pain was assessed using an 11-point numerical rating scale (NRS) pain score, where 0 indicated the absence of pain and 10 indicated the worst pain possible [12]. The patients were also classified according to the duration of preoperative pain (≤6 or > 6 months). The microscopic subinguinal varicocelectomy was performed under the influence of general anesthesia by three experienced surgeons by using a similar technique as described by Owen et al. [13], except for ligation followed by transection of the veins. All patients were asked to follow-up at 2 weeks, 3 months, and 1 year after surgery. Follow-up evaluation included physical examination, assessment of postoperative pain by 11-point NRS, and Doppler ultrasound to assess varicocele recurrence. Surgical success was defined as pain relief and a score of 0 on the 11-point NRS after the procedure. Failure was defined as the persistence of pain and a score of ≥1 on the 11-point NRS. The primary objectives were to assess the surgical success rate after microscopic subinguinal varicocelectomy and to determine the factors that could predict postoperative pain relief.

### Statistical analyses

Data were analyzed using SPSS version 20 (IBM, Armonk, NY, USA). Descriptive statistics were used to report the demographic and clinical characteristics. Continuous variables are presented as the mean with standard deviation or median. Categorical variables are expressed as absolute numbers with frequencies or percentages. One-way analysis of variance was used to compare the preoperative patient conditions and postoperative outcomes. Univariate analysis was performed using the Chi-square test and logistic regression analysis to evaluate the relationship between possible predictive factors and pain relief. *P* value < 0.05 were considered as significant.

## Results

**Fig. 2:**
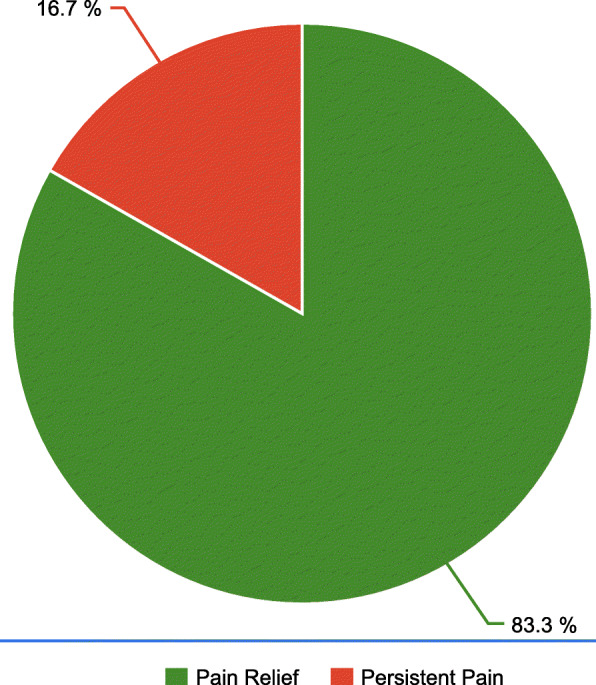
Outcomes of microscopic varicocelectomy for testicular pain

The present study included 132 patients. The average patient age at the time of surgery was 29.7 ± 6.6 years (range 18–26 years). Pain duration before surgery was 10.7 ± 4.5 months (range 3–18 months) with 85 (64%) patients describing the preoperative pain as dragging and 47 (36%) as dull pain. Bilateral varicocelectomy was performed in 48 (36.4%) patients and unilateral varicocelectomy in 84 (63.6%). No intraoperative complications were reported. Postoperative complications, including wound infection and hematoma, were documented in two (1.5%) patients. All patients were followed up (13.5 ± 4.9 months) with a range of 12–24 months. Of the 132 patients, 110 (83.3%) patients reported pain relief with a score of 0 on the 11-point NRS, whereas 22 (16.7%) reported persistent pain with either no change or very mild, insignificant change on the 11-point NRS for pain (mean points on 11-point NRS pain score, 5.05 ± 1.8 points) (Fig. 2). All 22 patients with persistent pain underwent Doppler ultrasonography, and none experienced varicocele recurrence. Univariate analysis showed that no preoperative parameter was predictive of pain resolution for patients who underwent varicocelectomy; however, microscopic varicocelectomy performed for unilateral testicular pain could significantly predict pain relief (*P* < 0.0001) (Table 1).

**Table 1:** Characteristics of 132 patients and factors that may predict pain relief

Variable	Pain relief group *n* = 110	Persistent pain group *n* = 22	***P*** value
**Mean age,** ± SD **(**years)	29.9 ± 8.1	28.5 ± 8.5	0.62
**Site of testicular pain**			
Unilateral	80	4	**< 0.0001**
Bilateral	30	18	0.083
**Preoperative pain quality**			
Dragging pain	70	15	0.81
Dull pain	40	7	0.74
**Preoperative pain duration**			
≤ 6 months	38	9	0.65
> 6 months	72	13	0.73
**Preoperative mean points on NRS**^**a**^ **pain score** ± SD	6.64 ± 1.3	6.73 ± 1.1	0.76
**Mean BMI**^**b**^, ± SD (kg/m^2^)	28.2 ± 1.2	28.4 ± 1.4	0.665
**Smoking**			
Yes	36	6	0.68
No	74	16	0.78
**Preoperative varicocele grade**			
I	20	8	0.74
II	52	18	0.98
III	62	20	0.84
**Mean of maximum vein diameter on ultrasound,** ± SD (mm)			
Left	4.9 ± 1.1	4.8 ± 1.6	0.80
Right	3.9 ± 1.0	4.2 ± 1.5	0.41
**Complications**	1	1	0.20

^a^numerical rating scale

^b^body mass index

## Discussion

Varicocelectomy is considered when conservative treatment fails to resolve varicocele-associated testicular pain [14]. All our patients had undergone conservative treatment for 3 months without any improvement before surgery. Currently, microscopic subinguinal varicocelectomy is the most common approach for the treatment of patients with varicocele [9]. Hence, we used this technique in the present study. Overall, 132 patients underwent microscopic subinguinal varicocelectomy for testicular pain. To the best of our knowledge, this is the second largest single-institution series that investigated the success rate and assessed the predictive factors of varicocelectomy for testicular pain [7]. The results of this study extend our knowledge of the true success rate of varicocelectomy for testicular pain, with a more specific definition of success than that reported in previous studies. Unilateral testicular pain (63.6%) was more common than bilateral pain (36.4%). Most patients (83.3%) reported complete resolution of pain. Only 16.7% experienced persistent pain, and none of them had recurrent varicocele, as examined using Doppler ultrasonography during follow-up, indicating that the primary cause of testicular pain was not varicocele and could be related to chronic orchialgia [6]. Microsurgical spermatic cord denervation has been described to treat chronic orchialgia; however, this procedure was not performed in the current study [15]. The success rate of varicocelectomy for testicular pain has varied among studies [10]. Our results corroborate those of the previous studies [5, 6, 8, 16–18], which reported postoperative symptomatic improvement and repair of painful varicocele in over 80% of patients. However, lower success rates of varicocelectomy for testicular pain were reported by Park et al. (52.8%) [10] and by Biggers and Soderdahl (48%) [19]. A lower rate was reported in another study, where 47.8% of the patients experienced complete resolution and 25.4% experienced partial resolution [14]. These variations in success rates could be a result of differences in the definition of success, surgical approach and techniques, or follow-up duration after surgery.

Only 1.5% of patients in our study reported postoperative complications, consisting of one wound infection and one hematoma. Reported predictors for postoperative pain resolution have included the varicocele grade and the quality and severity of preoperative pain [5, 8, 9, 20]. Kim et al. [20] stated that a significant number of patients who presented with dragging, dull, and aching pain experienced pain resolution after varicocelectomy. All our patient complaints matched these pain criteria but without significant correlation with pain relief, similar to those in other studies [6, 16]. In accordance with the finding of Karademir et al. [17], preoperative pain intensity and pain resolution were not correlated. However, Chen et al. [9] suggested that a preoperative pain score of > 6 could be predictive of symptomatic relief. Abd Ellatif et al. [6] reported no association between varicocele grade and pain relief after surgery. However, another study demonstrated that the preoperative grade of varicocele affected pain relief, where persistent pain was more common in patients with high-grade varicocele [8]. The duration of preoperative pain was another reported predictor of postoperative pain resolution [6, 10, 14, 16]. Abd Ellatif et al., Park et al., and Altunoluk et al. identified long pain duration before surgery (> 6, > ≥ 3, and > 3 months, respectively) as the only factor associated with pain resolution [6, 14, 16]. In contrast, in one study [10], a short preoperative pain duration of < 6 months predicted postoperative pain resolution. Our study showed an insignificant relation between pain relief and the duration of preoperative pain. Possible explanations for these variations in the duration of pain as a predictive factor are differences in pain duration criteria and the definition of success. Hence, further prospective randomized studies are required. Moreover, some studies have reported subinguinal ligation and microsurgical varicocelectomy as more effective in relieving varicocele-associated pain than other surgical techniques [20, 21]. However, in our study, we adopted only the gold standard microscopic subinguinal approach [13]. In another study, greater number of ligated veins (> 7) was a significant prognostic factor for pain relief after varicocelectomy [9]. No predictors for pain resolution were found, apart from the association of varicocelectomy for unilateral testicular pain with pain relief.

The limitations of this study include a relatively short median follow-up of just over 1 year and its retrospective design. Prospective randomized studies are needed to validate our findings. Given that patients with bilateral varicoceles have two different varicocele grades, there was an uneven distribution of patients for the varicocele grade, creating a potential for bias. In addition, not including other predictive factors such as various surgical techniques, varicocele location, and number of ligated veins could have potentially affected the pain resolution and success rate after varicocelectomy [9, 10, 21].

## Conclusions

Microscopic subinguinal varicocelectomy for testicular pain has high success (83.3%) and low complication (1.5%) rates when performed in selected patients after failed conservative treatment. Patients with unilateral testicular pain have a higher chance of pain relief after varicocelectomy. A prospective randomized study with a large sample size and long-term follow-up covering all the different prognostic factors for a painful varicocele is essential to validate the findings of the present study.

## Data Availability

The datasets used and/or analyzed during the current study are available from the corresponding author on reasonable request.

## Abbreviation

NRS: Numerical rating scale

## References

[1] Lomboy JR, Coward RM (2016). The varicocele: clinical presentation, evaluation, and surgical management. Semin Interv Radiol.

[2] Alsaikhan B, Alrabeeah K, Delouya G, Zini A (2016). Epidemiology of varicocele. Asian J Androl.

[3] Scaramuzza A, Tavana R, Marchi A (1996). Varicoceles in young soccer players. Lancet..

[4] Cho CL, Esteves SC, Agarwal AC (2019). Indications and outcomes of varicocele repair. Panminerva Med.

[5] Peterson AC, Lance RS, Ruiz HE (1998). Outcomes of varicocele ligation done for pain. J Urol.

[6] Abd Ellatif ME, Asker W, Abbas A, Negm A, Al-Katary M, El-Kaffas H (2012). Varicocelectomy to treat pain, and predictors of success: a prospective study. Curr Urol.

[7] Paick S, Choi WS (2019). Varicocele and testicular pain: a review. World J Mens Health.

[8] Yaman O, Ozdiler E, Anafarta K, Göğüş O (2000). Effect of microsurgical subinguinal varicocele ligation to treat pain. Urology..

[9] Chen SS (2012). Factors predicting symptomatic relief by varicocelectomy in patients with normospermia and painful varicocele nonresponsive to conservative treatment. Urology..

[10] Park HJ, Lee SS, Park NC (2011). Predictors of pain resolution after varicocelectomy for painful varicocele. Asian J Androl.

[11] Lyon RP, Marshall S, Scott MP (1982). Varicocele in childhood and adolescence: implication in adulthood infertility?. Urology..

[12] Farrar JT, Young JP, LaMoreaux L, Werth JL, Poole RM (2001). Clinical importance of changes in chronic pain intensity measured on an 11-point numerical pain rating scale. Pain..

[13] Owen RC, McCormick BJ, Figler BD, Coward RM (2017). A review of varicocele repair for pain. Transl Androl Urol.

[14] Park YW, Lee JH (2013). Preoperative predictors of varicocelectomy success in the treatment of testicular pain. World J Mens Health.

[15] Chaudhari R, Sharma S, Khant S, Raval K (2019). Microsurgical denervation of spermatic cord for chronic idiopathic orchialgia: long-term results from an institutional experience. World J Mens Health.

[16] Altunoluk B, Soylemez H, Efe E, Malkoc O (2010). Duration of preoperative scrotal pain may predict the success of microsurgical varicocelectomy. Int Braz J Urol.

[17] Karademir K, Senkul T, Baykal K, Ateş F, Işeri C, Erden D (2005). Evaluation of the role of varicocelectomy including external spermatic vein ligation in patients with scrotal pain. Int J Urol.

[18] Yeniyol CO, Tuna A, Yener H, Zeyrek N, Tilki A (2003). High ligation to treat pain in varicocele. Int Urol Nephrol.

[19] Biggers RD, Soderdahl DW (1981). The painful varicocele. Mil Med.

[20] Kim HT, Song PH, Moon KH (2012). Microsurgical ligation for painful varicocele: effectiveness and predictors of pain resolution. Yonsei Med J.

[21] Park JH, Pak K, Park NC, Park HJ (2019). How can we predict a successful outcome after varicocelectomy in painful varicocele patients? An updated meta-analysis. World J Mens Health.

